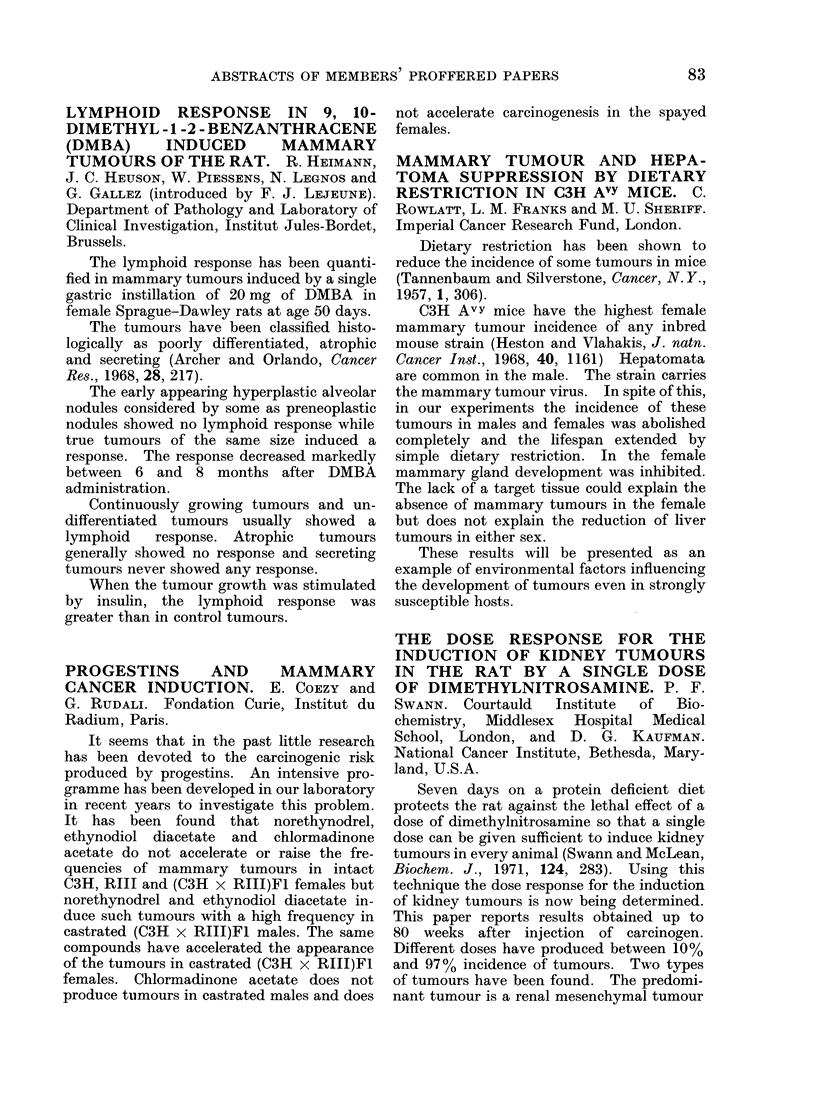# Mammary tumour and hepatoma suppression by dietary restriction in C3H Avy mice.

**DOI:** 10.1038/bjc.1973.98

**Published:** 1973-07

**Authors:** C. Rowlatt, L. M. Franks, M. U. Sheriff


**MAMMARY TUMOUR AND HEPATOMA SUPPRESSION BY DIETARY RESTRICTION IN C3H $A^{vy}$ MICE.** C. ROWLATT, L. M. FRANKS and M. U. SHERIFF. Imperial Cancer Research Fund, London.

Dietary restriction has been shown to reduce the incidence of some tumours in mice (Tannenbaum and Silverstone, *Cancer, N.Y.*, 1957, **1**, 306).

C3H $A^{vy}$ mice have the highest female mammary tumour incidence of any inbred mouse strain (Heston and Vlahakis, *J. natn. Cancer Inst.*, 1968, **40**, 1161) Hepatomata are common in the male. The strain carries the mammary tumour virus. In spite of this, in our experiments the incidence of these tumours in males and females was abolished completely and the lifespan extended by simple dietary restriction. In the female mammary gland development was inhibited. The lack of a target tissue could explain the absence of mammary tumours in the female but does not explain the reduction of liver tumours in either sex.

These results will be presented as an example of environmental factors influencing the development of tumours even in strongly susceptible hosts.